# CD 123 is a membrane biomarker and a therapeutic target in hematologic malignancies

**DOI:** 10.1186/2050-7771-2-4

**Published:** 2014-02-10

**Authors:** Ugo Testa, Elvira Pelosi, Arthur Frankel

**Affiliations:** 1 Department of Hematology, Oncology and Molecular Medicine, Istituto Superiore di Sanità, Viale Regina Elena 299, 00161 Rome, Italy; 2 UT Southwestern Medical Center, 5323 Harry Hines Blvd, Dallas, Texas 75396, USA

## Abstract

Recent studies indicate that abnormalities of the alpha-chain of the interleukin-3 receptor (IL-3RA or CD123) are frequently observed in some leukemic disorders and may contribute to the proliferative advantage of leukemic cells. This review analyzes the studies indicating that CD123 is overexpressed in various hematologic malignancies, including a part of acute myeloid and B-lymphoid leukemias, blastic plasmocytoid dendritic neoplasms (BPDCN) and hairy cell leukemia.

Given the low/absent CD123 expression on normal hematopoietic stem cells, attempts have been made at preclinical first, and then at clinical level to target this receptor. Since the IL-3R is a membrane receptor there are two relatively simple means to target this molecule, either using its natural ligand or neutralizing monoclonal antibodies. Recent reports using a fusion molecule composed by human IL-3 coupled to a truncated diphteria toxin have shown promising antitumor activity in BPDCN and AML patients.

## Introduction

The Interleukin (IL)-3, IL-5 and Granulocyte-Macrophage Colony Stimulating Factor (GM-CSF) Receptor, form a subfamily of membrane receptors, known as the Beta Common (β_c_) family of cytokines because these three receptors share the common signaling subunit β_c_ . Each of these three receptors is a heterodimer, composed by a cytokine-specific α subunit and a common β_c_ subunit, enabling high-affinity binding and cell signaling. These three cytokines play multiple biological functions, being involved in the control of normal and malignant hemopoiesis, native and adaptive immunity, and inflammatory response.

The structure, the function and main biological actvities of these three cytokines and their respective membrane receptors are covered by several recent excellent reviews, to which the reader is referred [1-4].

IL-3 is a pleiotropic cytokine, mainly produced by activated T-lymphocytes, regulating the function and production of hematopoietic and immune cell [5]. This cytokine was originally termed multi-colony stimulating factor (multi-CSF) for its property to stimulate the development of a wide-range of hematopoietic cells from bone marrow, including basophils, neutrophils, eosinophils, macrophages, erythroid cells, megakaryocytes, and dendritic cells [6,7]. The biologic activity of IL-3 is not limited only to the hematopoietic system, but it extends also to the endothelial lineage, where IL-3 acts as a stimulator of endothelial cell proliferation [8].

As above stated, the IL-3R is a heterodimer composed by alpha and beta chains. The IL-3Rα chain is a glycoprotein of 360 aminoacidic residues, composed by an extracellular domain of 287 amino acid residues, involving a predicted Ig-like domain, two FnIII domains, a transmembrane domain of 30 amino acid residues and by an intracellular domain of 53 residues [9-12].

### CD123 expression on human hematopoietic stem/progenitor cells

Many studies have explored the expression of CD123 at the level of repopulating stem cells and of various subpopulations of hematopoietic progenitor cells. In this context, CD123 expression at the level of CD34^+^ cells isolated from various sources of hematopoietic cells (fetal liver, cord blood, bone marrow and peripheral blood) was studied. Sato an coworkers showed that a part of human and cord blood CD34^+^ cells express CD123 and the growth of these cells in the presence of cytokines stimulating their proliferation resulted in an increased CD123 expression [13]. Accordingly, it was suggested that the primitive population of HPCs expressed low or absent CD123 levels [13]. Wognum et al provided evidence that early primate HPCs identified as CD34^+^/HLA-DR^dull^ cells express low levels of CD123, while CD34^+^ cells with negative or high CD123 expression were committed erythroid and myeloid progenitors, respectively [14]. Huang and coworkers have defined three subsets of CD34^+^ cells according to the level of surface CD123: CD34^+^CD123^bright^ cells were myeloid and B-lymphoid progenitors, whereas the erythroid progenitors were mainly contained in the CD34^+^CD123^negative^ subset; CD34^+^CD123^low^ cell subset contained a heterogeneous population of early and committed progenitor cells [15]. More recently, Manz and coworkers have subfractionated the CD34^+^CD38^+^ cell population, isolated either from bone marrow or cord blood, according to the positivity for CD123 and CD45RA and have shown that: IL-3Rα^low^CD45RA^+^ cells mainly contained granulo-monocytic progenitors (GMP); IL-3Rα^-^CD45RA^-^ cells mainly contained erythroid and megakaryocytic progenitors (MEP); IL-3Rα^low^CD45RA^-^ cells give rise to both GMPs and MEPs and contained the progenitors of both populations [16].

CD123 expression during hemopoietic differentiation was explored by Testa et al, providing evidence that this receptor was expressed on the majority of CD34^+^ hemopoietic progenitors and its expression is rapidly lost during erythroid and megakaryocytic differentiation, moderately decreased during monocytic differentiation and was sustained in the granulocytic lineage [17]. The IL-3R/GM-CSFR/IL-5R β_c_ was expressed at low levels in CD34^+^ cells and its expression increased during the initial stages of granulocytic and monocytic differentiation and was maintained up to the late stages of maturation, while it was transiently expressed in the erythroid and megakaryocytic differentiation only during the early stages of differentiation and then disappeared [18].

CD123 expression on normal hematopoietic stem cells (HSCs) was addressed in some studies. Taussig and coworkers have isolated CD34^+^/CD38^-^ cells from cord blood and bone marrow: this cell fraction contains HSCs, defined as cells capable of long-term repopulation of irradiated SCID mice [19]. In cord blood, the majority of CD34^+^/CD38^-^ cells expressed CD123, as well as other myeloid antigens such as CD13 and CD33; in bone marrow, the percentage of CD123^+^ cells among CD34^+^/CD38^-^ cells was lower [19]. In a second set of experiments, CD34^+^/CD38^-^CD123^+^ and CD34^+^/CD38^-^ CD123^-^ cells were separated, providing evidence that SCID-repopulating cells were more frequent among CD123^+^ than among CD123^-^ cells [19].

Jin and coworkers used another approach using a neutralizing anti-IL-3Rα mAb (7G3); *ex vivo* treatment of CD34^+^ bone marrow cells elicited a moderate inhibitory effect on the SCID-repopulating capacity (a decrease of about 30% of the engrafting capacity was observed following incubation of bone marrow cells with this antibody). These observations indicate that only a part of normal HSC express CD123 and therefore the majority of them should be not affected by a therapy targeting this receptor [20].

### CD123 in blastic plasmocytoid dendritic cell neoplasm

Blastic plasmocytoid dendritic cell neoplasm (BPDCN), formerly known as blastic natural killer cell lymphoma CD56^+^/CD4^+^ hematodermic neoplasm, is a rare hematologic malignancy derived from the uncontrolled growth of plasmocytoid dendritic cells. The nomenclature of BPDCN was adopted in 2008 by the World Health Organization (WHO) classification after the analysis of its main immunophenotypic, molecular and functional properties, such as pronounced CD123 expression, TCL1 expression and production of type I IFN, all suggesting a close link and a pathogenetic relation to the plasmocytoid dendritic cell lineage. After many years of intensive studies various subsets of human dendritic cells have been identified, distinguished according to their cellular origin, phenotypic, molecular and functional properties: (a) myeloid dendritic cells, expressing typical myeloid antigens; (b) plasmacytoid dendritic cells (PDC), lacking the expression of mnyeloid antigens and characterized by the high expression of CD123; (c) CD14^+^ dendritic cells, known also as interstitial dendritic cells, derived from classical monocytes; (d) Langherans and microglia cells, two types of specialized dendritic cells, the former ones capable of generating migratory dendritic cells and the latter ones considered as tissue specialized macrophages. PDCs are major type I interferon-producing cells and play an important role in antiviral immunity and induction of immunologic tolerance.

The study of CD123 was of fundamental importance for the identification and for the study of human plasmacytoid dendritic cells. Thus, Olweus and coworkers reported in 1997 the identification in a subset of dendritic cells in the T cell-dependent areas of human lymphoid organs of cells characterized by the high expression of CD123; these cells are originated from CD34^+^/CD123^high^ progenitors [21]. In 2002, MacDonald and coworkers reported the identification of a subset of dendritic cells in human peripheral blood characterized by the very high expression of CD123 [22].

In the study of BPDCN patients it was of fundamental importance the discovery by Chaperot and coworkers about the expression of IL-3Rα chain on CD4^+^/CD56^+^ leukemic cells: upon *in vitro* incubation with IL-3 the leukemic cells undergo a partial maturation and became a powerful inducer of naïve CD4^+^ T cell proliferation [23]. In contrast, the GM-CSFRα chain (CD116) expression was absent/low on these leukemic cells [24]. Upon growth *in vitro* in the presence of IL-3 leukemic cells derived from BPDCN patients increased the expression of molecules associated with antigen presentation, such as CD1a and CD40, and displayed a consistent level of antigen-presenting ability to allogeneic lymphocytes in mixed lymphocyte cultures [25]. These observations indicate that leukemic cells in BPDCN may act as potent antigen cells *in vivo*, and this property is controlled by IL-3 [25].

The high expression of CD123 observed on BPDCN cells is not surprising in view of the expression of this receptor also in the normal cellular counterpart. In fact, immature plasmacytoid dendritic cells express IL-3Rα. Importantly, IL-3 is required for an efficient *in vitro* generation of plasmacytoid dendritic cells [26]. Thus, Buelens and coworkers showed that monocytes cultured with IL-3 and IFN-beta give rise to a population of dendritic cells displaying many properties of plasmacytoid DCs and highly expressing CD123. A more recent study provided evidence that IL-3 cooperates with FLT3 Ligand and TPO to induce the efficient production of plasmacytoid dendritic cells displaying phenotypic and functional properties of plasmacytoid dendritic cells [27].

Patients with BPDCN have a poor prognosis when treated with conventional standard chemotherapy. The only treatment capable of inducing durable remissions is the high-dose therapy followed by allogeneic-stem cell transplantation (allo-SCT); in this setting, graft-versus-malignancy effects can contribute to BPDCN control after allo-SCT [28]. In a recent study representing the largest series of these patients (43 patients) never reported in the literature, it was observed a median overall survival of about 8-9 months following treatment with standard chemotherapeutic regimens used for AMLs and ALLs; the median overall survival of patients undergoing allogeneic stem cell transplantation was about 23 months [29].

Given the high IL-3Rα expression observed on CD4^+^CD56^+^ leukemic cells and their poor response to standard therapy it seemed important to evaluate a possible therapeutic approach through the targeting of this membrane receptor. Thus, Frankel and coworkers have recently reported the treatment of seven BPDCN patients (some heavily pretreated) with SL-401, an agent targeting the IL-3Rα (this agent is composed by human IL-3 coupled to a truncated diphtheria toxin payload). A single cycle of 5-days treatment with this agent at 12.5mcg/kg IV over 15 minutes elicited a pronounced anti-tumor response inducing clinical remissions in most patients lasting several months to over a year [30]. These preliminary promising observations represent the basis for future studies involving multiple cycles of treatment and the enrolling in the future of less heavily treated patients.

### CD123 in hairy cell leukemia

CD123 expression was explored in B lymphoproliferative disorders of mature B-lymphocytes. An initial study was based on the analysis of 122 patients with lymphoproliferative disorders of mature B-lymphocytes, showing that the large majority of chronic lymphocytic leukemias and mantle and follicular lymphomas were CD123 negative, while CD123 was constantly expressed on hairy cell leukemia cells [31]. Del Giudice and coworkers have explored in detail the expression of CD123 in neoplastic cells derived from 59 patients with B-cell disorders with circulating hairy or villous lymphocytes: CD123 was expressed on hairy cell leukemia, but not on the variant form of hairy cell leukemia and splenic lymphoma with villous lymphocytes [32]. A more recent study confirmed the importance of CD123 to differentiate hairy cell leukemia from hairy cell leukemia-variant: in fact, while HCLs strongly express CD123, the expression of this membrane receptor was absent or low/very low on HCL-variant cells [33]. Other criteria help to distinguish these two similar diseases, such as the morphology, the positivity for annexin A1 and the presence of the BRAFV600E mutation [33]. Another recent study showed that the positivity for CD123 was essential for distinguishing both HCL and HCL-variants from other B-cell chronic lymphoproliferative disorders; furthermore, the positivity for CD123 was essential for distinguishing HCL from HCL-variant [34].

### CD123 in acute lymphocytic leukemias

An initial study by Munoz and coworkers, based on the analysis of a small number of patients, provided evidence that B-ALL express CD123, while T-ALLs were CD123-negative [31]. Importantly, in this study these authors have explored also normal bone marrow CD19^+^CD33^-^CD10^+^ B-lymphoid precursors and have observed that these cells are CD123-negative [31]. Testa and coworkers have confirmed these observations [35].

In 2009, Djokic and coworkers reported a detailed analysis of CD123 expression on B-ALLs. Particularly, these authors analyzed 95 pediatric and 24 adult B-ALLs, showing that in 31% of cases B-ALL blasts displayed strong CD123 expression, in 61% moderate expression and in 8% of cases were CD123-negative [28]. In contrast, early B-cell precursors, intermediate B-cells and mature B-lymphocytes displayed only absent or low CD123 expression [36]. The correlation between CD123 expression and molecular B-ALL subtypes showed several interesting findings: the CD123 expression in B-ALLs correlated with hyperploid genotype, a frequent genetic abnormality in childhood ALLs; in contrast, B-ALLs associated with other genetic abnormalities, such as ETV6/RUNX1, BCR/ABL1, PBX1/TGF3 translocations or a normal karyotype, did not display CD123 overexpression, compared to normal B-cell precursors [36]. Hassaneien and coworkers have confirmed these findings and, particularly, have analyzed the normal compartment of B-cell precursors, as well as B-ALL leukemic blasts [37]. Particularly, these authors have shown that early B-cell CD3^+^ precursors do not express CD123, while more mature CD34^+^ B-cell elements display the CD123 expression. In B-ALLs in 89% of cases CD123 expression was observed: in 80% of cases CD123 expression was associated with CD34 expression; in 9% of cases ALL blasts express CD123, but not CD34 [37]. According to these observations, these authors concluded that while in normal B cell precursors a discordant pattern of CD123 and CD34 antigen expression was observed, in B-ALL blasts a concordant pattern of expression of these two antigens was observed [37]. Interestingly, in the majority of B-ALL patients, CD123^+^/CD34^+^ cells were observed post-chemotherapy [37]. In a more recent study Coustan-Smith and coworkers carried out an analysis on 270 patients with newly diagnosed childhood ALL based on the analysis by flow cytometry of 30 membrane antigens, including CD123 [38]. Of these 30 markers, 22 were differentially expressed in ALL cases compared to CD19^+^CD10^+^ B-cell progenitors; particularly, CD123 was markedly more expressed in the majority of cases on B-ALL blasts, compared to normal B cell progenitors [38]. Some of these markers, including CD123 were shown to be very useful fo the detection of minimal residual disease, even at the level of the definition of a very minor residual lymphoid leukemic cell population [38]. It is important to underline that this study based on the analysis of a large number of B-ALL samples allowed also to define the ALL genetic subtypes exhibiting the highest CD123 expression. This analysis unequivocally allowed to establish that hyperdiploid B-ALLs (51-65 chromosomes) highly expressed CD123 [38]. CD123, together with CD86, CD97 and CD200 (all overexpressed) may be used for a tentative flow cytometric identification of hyperdiploid B-ALLs [38]. The very pronounced CD123 expression in hyperdiploid B-ALLs offers the opportunity for a possible therapeutic targeting [38].

### CD123 in Acute Myeloblastic Leukemia (AML)

CD123 expression in AML was explored in detail and these studies have recently led to the therapeutic targeting of this receptor. Initial studies in AMLs have defined an aberrant overexpression of CD123 on CD34^+^CD38^-^ AML cells, while the normal bone marrow counterpart CD34^+^CD38^-^ does not express CD123 [39]. To assess the functional role of CD34^+^CD38^-^CD123^+^ cells these cells were purified from AML samples and injected into immunodeficient mice: these cells were able to initiate and maintain the leukemic process into immunodeficient mice and, therefore, act as leukemic stem cells [39]. In a subsequent study Munoz and coworkers have performed a screening on IL-3Rα expression in hemolymphopoietic malignancies provided evidence that this membrane receptor is very frequently expressed in B-ALL and AML [31].

Given these initial observations, Testa and coworkers have explored a large number of AMLs and ALLs with the particular aim of defining a possible overexpression of this membrane receptor compared to the normal counterpart (i.e., normal CD34^+^ cells), showing that IL-3Rα was overexpressed in 45% of AML patients [35].

Subsequent studies on IL-3Rα in AMLs have been performed with the specific aim of defining the possible biologic effects induced by IL-3Rα overexpression in leukemic blasts, the AML subsets particularly associated with the receptor hyperexpression and the receptor expression on leukemic stem cells.

Testa and coworkers have explored the possible effects induced by CD123 overexpression at the level of leukemic cells and have shown that: (a) leukemic blasts overexpressing IL-3Rα exhibit higher cycling activity and increased resistance to apoptotic triggering elicited by growth factor deprivation; (b) AMLs overexpressing IL-3Rα frequently display constitutive Stat5 phosphorylation; (c) the incubation of AML blasts displaying high IL-3Rα expression with IL-3 induced Stat5 activation at significantly higher levels than in leukemic cells with normal IL-3Rα levels [35]. Importantly, IL-3Rα overexpression on AML blasts was associated with increased cellularity at diagnosis and with a negative prognosis [35].

Other studies were focused to the identification of AML subsets associated with IL-3Rα overexpression. In this context, initial observations by Testa and coworkers showed AMLs expressing high levels of CD123 displayed some peculiar immunophenotypical features [40]. Subsequent studies have shown that AMLs overexpressing IL-3Rα are frequently associated with AMLs displaying FLT3-Internal Tandem Duplication (FLT3-ITD) mutations and are associated with some frequent immunophenotypical features consisting in low CD34 expression and high CD11b and CD14 expression [41]. Importantly, in these AMLs the high CD123 expression well correlated with CDw131 (IL-3R β_c_) and CD116 (GM-CSFRα) expression, thus suggesting in these AMLs the concomitant overexpression of functional IL-3R and GM-CSFR. These AMLs were also characterized by the co-expression of receptors for endothelial growth factor [41]. Interestingly, high IL-3Rα levels were also observed in a subgroup of AML patients characterized by FLT3 overexpression, not associated with mutations of this receptor [42]. According to these two studies it was suggested that in the majority of AMLs overexpressing CD123 the enhanced and deregulated signaling originated by IL-3R and FLT3 contribute to provide a survival and growth advantage to leukemic AML blasts [42]. These findings were conformed in a more recent study. In fact, Rollins-Raval have shown that CD123 overexpression was observed in 83% of FLT3-ITD-mutated AMLs [43]. Furthermore, 62% of AML cases with nucleophosmin mutations displayed CD123 overexpression [43]. Gonen and coworkers have identified a subgroup of AMLs, pertaining to the cytogenetically intermediate-risk group, characterized by CD25 (IL-2R) positivity and by a poor prognosis: these AMLs display CD123 overexpression and frequently have FLT3-ITD mutations [44].

Many studies were devoted to the analysis of IL-3Rα expression in leukemic stem cells (LSCs). As above mentioned, an initial study by Jordan and coworkers provided evidence that CD34^+^/CD38^-^ purified leukemic cells, enriched in LSCs, overexpress in the majority of AMLs CD123. CD123^+^/CD34^+^ leukemic cells were able to initiate a leukemic process when transplanted into immunodeficient mice [45]. The CD123 was then used in other studies as a marker to isolate LSC populations from AML samples and to provide a characterization of these cells and it was shown that these cells display a constitutive activation of NF-kB [45]. Putative CD34^+^/CD38^-^/CD123^+^ LSCs were detectable in about 75% of AMLs [46] and their number is predictive of the clinical outcome [47]. In fact, a proportion of CD34^+^/CD38^-^/CD123^+^ cells greater than 15% in AML patients with unfavourable karyotype was associated with a lack of complete remission; furthermore, the presence of more than 1% of CD34^+^/CD38^-^/CD123^+^ cells had a negative impact on disease-free survival and overall survival [47].

Other recent studies have provided evidence that CD123 is a useful marker of leukemia-initiating cells in Fanconi anemia AML cells. In fact, Du and coworkers have isolated CD34^+^/CD38^-^ cells from three Fanconi anemia patients: Starting from these cells IL-3Rα^+^ and IL-3Rα^-^ subpopulations and only the positive population was able to initiate the development of a leukemic process when inoculated into immunodeficient mice [48].

The definition of treatment response criteria is of fundamental importance for the development of more efficacious therapeutic strategies for AMLs, but these criteria remained virtually unchanged and the definition of “complete remission” remained still basically the same of many years ago [49]. In spite these limitations, several clinical trials have shown that high-sensitivity measurement of residual disease burden (minimal residual disease, MRD) during and after treatment can be performed and may be used to try to improve clinical outcomes by guiding to additional clinical interventions of some patients in complete clinical remission [49]. The measument of MRD represents already the standard of care for CML and APL, but it could be extended also to non-APL AMLs. The evaluation of MRD on these AMLs is mainly related to the detection of leukemia-specific genetic abnormalities. However, the analysis of MRD in AML patients could be also performed through the monitoring by flow cytometry of leukemic cell populations. Recent studies suggest that the levels of expression of CD123 and of other membrane antigens represent a strong prognostic marker for relapse. Recent studies carried out by Larsen and coworkers have identified the human Myeloid Inhibitory C type-like lectin (hMICL) as a stable and reliable AML antigen expressed in 90% of AML samples, as well as CD123 [50]; importantly, the expression of these two antigens remained constant in paired samples at diagnosis and at relapse [50]. In a second study the same authors provided evidence that the detection of these two membrane markers could represent a very useful tool as a Minimal Residual Disease (MRD) prognostic biomarker. The detection of high levels of CD123^+^/hMCL^+^ cells at post-induction time-point represent a strong prognostic marker for relapse in patients in hematological complete response [51]. Importantly, in post-induction AML patients CD123^+^/hMCL^+^ levels strongly correlate with the level of suitable molecular markers detected by qPCR [43]. Other recent studies have addressed the potential utility of the detection of CD123^+^/hMCL^+^ cells for the monitoring and for the study of MRD in FLT3-ITD^+^ AML patients. A first study clearly showed that the existence of high FLT3-ITD allelic burdens in CD123^+^/hMCL^+^ FLT3-ITD^+^ AML blasts at diagnosis, thus indicating that these cells are malignant [52]. A second study provided evidence that CD123 expression predicts minimal residual disease and relapse in AML patients with FLT3-ITD mutations [53]. CD123 expression in FLT3-ITD^+^ AMLs at the level of a cell population CD34^+^CD38^-^CD99^+^ enriched in leukemic stem cels was clearly higher that detectable on the same cell population isolated from normal bone marrow; interestingly, these CD123^+^ leukemic cells exhibited an increased level of cell signaling activity along the Stat5 and PI3K/AKT pathways [53].

### Targeting of IL-3R in acute leukemias

All these findings have suggested that IL-3R may represent a potentially important target for the development of new antileukemic drugs [40]. Since the IL-3R is a membrane receptor there are three means to target this molecule, either using its natural ligand or specific monoclonal antibodies or a small molecule inhibiting the receptor signaling.

In this context, initial studies were focused to develop a IL-3R targeting agent besed on the natural ligand IL-3. To this end, a genetically engineered fusion toxin composed of the first 388 amino acid residues of diphteria toxin (DT) with a His-Met (H-M) linker was fused to human IL-3 [54]. This DT_388_IL-3 was shown to be toxic for leukemic blasts [55] and *in vivo* studies have shown that it is well tolerated up to 100 ug/Kg [56,57]. Importantly, the rate of cell killing of leukemic blasts induced by DT_388_IL-3 was significantly correlated with the level of IL-3Rα/IL-3Rβ expressed on leukemic blasts [57-59]. It is important to note that the DT_388_IL-3 fusion protein was found to be toxic toward leukemia-initiating cells [58]. Interestingly, the fusion of the DT to a variant of IL-3 with increased binding affinity (IL-3[K116W]) resulted in a fusion protein, DT_388_IL-3[K116W] significantly more active than DT_388_IL-3 in mediating leukemic cell killing [55]. Interestingly, DT_388_IL-3[K116W] was clearly more active than DT_388_IL-3 in mediating the killing of leukemic progenitor cells [59].

A second approach to target the IL-3R in leukemic cells is represented by the use of a specific monoclonal antibody. In this context, a neutralizing anti-IL-3R could be a perfect reagent for its properties to neutralize IL-3R from one side and to activate innate immunity from the other side. A reagent corresponding to these properties is represented by the anti-CD123 mAb 7G3 capable of inhibiting IL-3-mediated proliferation of leukemic cell lines [60]. It was shown that the targeting of CD123 using the 7G3 mAb impairs leukemic stem cells *in vivo*[20]. This effect seems to be related to two different mechanisms: one dependent upon inhibition of homing and engraftment of leukemic CD34^+^/CD38^-^ cells into immunodeficient mice; the other related to the activation of innate immunity into NOD/SCID mice [20]. It is important to note that the inhibitory effects of the 7G3 mAb are not restricted only to the CD34^+^/CD38^-^ leukemic subpopulation, but are exerted on the whole bulk leukemic cell population [20].

Using the 7G3 mAb a construct was developed linking to this antibody a nuclear traslocation sequence (NLS) and (111)In [(111)In-NLS-7G3] [61]. This modified mAb was able to bind to AML cells, to be internalized and to be imported into the nucleus [61]. Using AMl cells labeled with this modified antibody it was possible to determine the sites of engraftment of leukemic cells into immunodeficient mice [61].

The 7G3 monoclonal antibody was humanised and affinity-matured and it was engineered at the level of the Fc-domain to optimise potential cytotoxicity against AML cells: the resultant antibody, CSL362, retained the ability to neutralise IL-3 and exhibited enhnaced affinity for the FcgammaRIIIa (CD16) on NK cells [62]. *In vitro* and *in vivo* studies supported a greater activity of CSL362, compared to the native 7G3 mAb, against CD123^+^ leukemic cells [60]. Both whole leukemic AML blasts and CD34^+^CD38^-^CD123^+^ leukemic stem cells were sensitive to CSL362-induced ADCC [62]. The isolation of this antibody prompted its preclinical exploration in view of future clinical studies. Pre-clinical studies to evaluate the pharmacokinetic and pharmacodynamic properties of CSL362 have been carried out in non-human primates [63]. These studies provided evidence for that it concerns pharmacokinetics about a pharmacokinetic profile similar to that previously reported for other humanised antibodies and for that concerns pharmacodynamics about a marked dose-dependent depletion of peripheral blood basophils and plasmacellular dendritic cells, associated with a transient decrease of NK lymphocytes and with virtually no effects at the level of monocytes and pluripotent bone marrow progenitor/stem cells [63]. These pharmacodynamic studies were completed by the observation that CSL362 administration to NSG mice xenografted with human primary AML cells considerably potentiated the antileukemic effect elicited by the administration of the drug combination cytabarine/daunorubicin [64]. Interestingly, a recent study provided evidence that the CSL362 antibody seems to be able to induce the killing of leukemic stem cells in chronic myeloid leukemia (CML). In fact, Nievergall and coworkers have shown that CD123 is overexpressed in CD34^+^CD38^-^ CML cells, compared to normal CD34^+^CD38^-^ cells: these levels of CD123 expression were higher both in chroinic phase and blast crisis CML patients, with levels increasing upon disease progression [65]. CSL362 was able to target and to kill CD123^+^ CML leukemic stem cells and markedly reduced engraftment of CML cells into NSG mice [65]. It is of interest to note that in mice treated with CSL362 ADCC-facilitated lysis of CD123^+^ leukemic cells was mediated not by mouse NK cells, but also by the few CML patients’ autologous NK cells [65]. Importantly, CSL362 neutralized IL-3-mediated rescue of tyrosine kinase inhibitor (TKI)-induced cell death and potentiated the effect of TKIs at the level of CML progenitors [65].

Recently, Kuo and coworkers have reported the development of a bifunctional fusion anti-CD123 and anti-CD3 antibody (CD123xCD3 bispecific scFv). This fusion antibody exhibits several interesting properties: (a) it exhibits increased target cell-binding affinity; (b) it possesses increased stability due to increased serum half-life; (c) it is able to achieve T-cell-mediated target cell killing [66]. More recently, Hussaini and coworkers have reported the development of a bispecific antibody, containing V^H^ of one antibody in tandem with the V^L^ of the other antibody and interacting with the N-terminal extracellular domain of human CD123 and to the extracellular domain of CD3 in the human T cell receptor complex [67]. The incubation of this CD3xCD123 DART with leukemic AML blasts resulted in a marked activation and proliferation of T cells present in these cells and in the killing of leukemic cells [67]. The infusion of DARTs to AML xenografts into NSG mice resulted in an almost complete clearance of leukemic cells in peripheral blood and in subtotal inhibition of leukemic cells in bone marrow [67].

Another recent study reported the development of a novel conjugate of single-chain Fv antibody fragments specific for CD123 with an anti-CD16 antibody. This fusion antibody exerts a potent lysis of leukemic cells [68]. Through the anti-CD16 moiety this fusion antibody is able to bind NK lymphocytes and monocytes/macrophages [68]. The same authors reported the construction of a recombinant trispecific single-chain Fv derivative directed against CD123, CD33 and CD16, mediated effective elimination of AML cells, including leukemic stem cells [69].

Finally, Tettamanti and coworkers have reported a different approach for targeting CD123 based on the transduction of cytokine-induced killer (CIK) cells with a retroviral vector encoding an anti-CD123 chimeric antigen receptor [70]. Transduced CIK cells were able to kill AML blast cells, including leukemic stem/progenitor cell populations [70]. Another recent study reported the development of two Chimeric Antigen Receptors (CARs) containing a CD123 specific ScFv in combination with a CD28 costimulatory domain and CD3-related signaling domain, targeting different epitopes of CD123 [71]. CD123 CAR redirected cytotoxic T cells exerted potent effector activity *in vitro* and *in vivo* against CD123^+^ AML cells [71]. Importantly, T cells derived from AML patients were modified to express CD123 CARs and were shown to be able to lyse autologous AML blasts *in vivo*[71]. Furthermore, Gill and coworkers reported the development of a CAR consisting a CD123-specific ScFv-derived from hybridoma clone 32716 and signaling domains from 4-1BB (CD137) and TCR-ζ [72]. CART cells incubated in the presence of primary AML blasts undergo proliferation, release of inflammatory cytokines and induced the killing of leukemic cells; in NOD-SCID-IL2Rγ^-/-^ (NSG) mice engrafted with either leukemic cell lines or primary leukemic blasts, CART 123 treatment induced the eradication or a marked inhibtion of leukemic growth, respectively; CART 123 immunotherapy of NSG mice reconstituted with normal human CD34^+^ cells resulted in a non-complete eradication of human bone marow cells [72]. These observations have suggested that CART 123 could represent a novel valuable conditioning regimen prior to hematopoietic cell transplantation [72].

Some of these compounds entered phase I clinical studies (Table 1) aiming to define the safety profile and to have the first indications about a possible therapeutic impact of CD123 targeting in patients with advancer AMLs. Thus, the fusion molecule composed by human IL-3 fused to a truncated diphteria toxin payload (the compound was called SL-401) was introduced in a phase I clinical trial aiming to evaluate its safety and therapeutical impact in a group of AML patients heavily pretreated [73]. In this study, SL-401 demonstrated the induction of objective clinical responses, including 2 durable complete responses and 5 partial responses and improved the overall survival [73]. Furthermore, the SL-401 administration was well tolerated [73]. The same compound SL-401 was evaluated also in the context of a phase I clinical study involving eight patients with blastic plasmocytoid dendritic cell neoplasms [30]. In this group of heavily pretreated patients with advanced disease the treatment with a single cycle of SL-401 was well tolerated and demonstrated prominent antitumor activity, with most of patients achieving a complete response [30].

**Table 1:** Agents used for the targeting of CD123 in leukemic patients

**Agent**	**Efficiency in treatment model**	**Targeting of LSCs**	**Effect on normal**	**Postulated mechanism**	**Clinical studies**
SL-401 (human IL-3 conjugated to a truncated diphteria toxin)	Induces a pronounced clearing of leukemic cells, including AML blasts, BPDCN tumor cells, MM cells	Induces killing of CFU-L. Induces the killing of CD34^+^/CD38^-^ leukemic cells	40-50% inhibition of CFU-GM	Induces the apoptotic killing of cells expressing CD123	Phase I in AMLs and BPDCN. Phase II studies are planned
CSL362 (humanized form of the 7G3 anti-CD123 mAb, IL3 neutralizing)	Reduces AML leukemic stem cell homing, engraftment and self-renewal ability and improves the survival of mice, with minimal effect on normal BM cells	Inhibits spontaneous and IL3-induced proliferation of CD34^+^/CD38^-^ leukemic cells. In vivo treatment of xenografted NOD/SCID mice targets LSCs	Dose-dependent depletion of PB basophils and plasmacellular dendritic cells, transient decrease of NK cells and no effect on monocytes and HPCs and HSCs.	ADCC-facilitated lysis of leukemic CD123^+^ leukemic cells mediated by NK cells	Phase I in AMLs
CD123 CART cells (T cells expressing CD123-specific receptors)	Induces an antileukemic effect in nude mice xenografted with human CD123^+^ AML cells	70-80% inhibition of CFU-L	40-50% inhibition of CFU-GM; 10-20% inhibition of BFU-E. The cytotoxic effect is limited to cells displaying high CD123 expression	Antibody-mediated T cell killing of CD123^+^ leukemic cells. Activation of cytokine production and effector functions of T lymphocytes	None

Other phase I studies involved the use of monoclonal antibodies anti-CD123. CSL360 is a recombinant chimeric mAb that binds to IL-3Rα at the level of the same epitope recognized by the 7G3 mAb. 40 patients with relapsed, refractory or high-risk AML were treated with intravenous infusions of this antibody ranging from 0.1 to 10 mg/kg. The patients did not show any significant response to the treatment, with exception of one patient achieving a complete response [74]. Another phase I study of CSL362 (anti-IL-3Rα/CD123 monoclonal antibody) in patients with CD123^+^ acute myeloid leukemia in complete remission or complete remission with incomplete platelet recovery at high risk for early relapse is ongoing (NCT01632852, Clinical Trials Gov).

In the next decade, advances in patient selection and drug design should establish a role for CD123 targeted therapy in hematologic malignancies.

## Conclusions and future directions

In conclusion, the strudies carried out during the last two decades have clearly shown that CD123 is overexpressed in many hematologic malignancies, including BPDCNs, AMLs, B-ALLs, Hairy Cell Leukemia. Importantly, in some of these conditions, such as AMLs, the high expression of CD123 is a feature of leukemic stem cells, making very attractive the opportunity to therapeutically target this membrane receptor. Therefore, various agents have been developed in an attempt to use CD123 targeting to kill leukemic cells overexpressing this receptor, either using a fusion protein formed by recombinant IL3 fused to diphteria toxin (SL401) or a humanized neutralizing anti-CD123 monoclonal antibody or T cells transduced withg a retroviral vector encoding an anti-CD123 chimeric antigen receptor. Some of these compounds were introduced in clinical studies in the context of phase I studies in AML and BPDCN patients with promising results. Additional studies will be required to assess the role of these CD123-targeting drugs, not only when used alone, but also in combination with standard anti-leukemic drugs in the treatment of various leukemic cionditions. Furthermore, it would be particularly interesting to assess the possible therapeutic impact of CD123-targeting drugs to attempt the treatment of minimal residual disease.

## Competing interests

The authors declare that they have no competing interests. Arthur E. Frankel has proprietary interests in SL-401 or DT388IL3 and the clinical study of SL-401 is funded by Stemline Therapeutics.

## Authors’ contributions

The concept of this paper was devised by UT, EP and AEF. All these three authors contributed to the intellectual input of the paper. All authors read and approved the final manuscript.
